# Data on metabolic profile of insulin-degrading enzyme knockout mice

**DOI:** 10.1016/j.dib.2019.104023

**Published:** 2019-05-24

**Authors:** Diego O. Borges, Maria João Meneses, Tânia R. Dias, Fátima O. Martins, Pedro F. Oliveira, Marco G. Alves, M. Paula Macedo

**Affiliations:** a CEDOC – Centro de Estudos de Doenças Crónicas, NOVA Medical School/Faculdade de Ciências Médicas, Universidade Nova de Lisboa, Lisboa, Portugal; b Department of Microscopy, Laboratory of Cell Biology and Unit for Multidisciplinary Research in Biomedicine (UMIB), Abel Salazar Institute of Biomedical Sciences (ICBAS), University of Porto, Porto, Portugal; c ProRegeM PhD Programme, NOVA Medical School/Faculdade de Ciências Médicas, Universidade Nova de Lisboa, Lisboa, Portugal; d I3S - Instituto de Investigação e Inovação em Saúde, University of Porto, Porto, Portugal; e Department of Genetics, Faculty of Medicine, University of Porto, Portugal; f Portuguese Diabetes Association - Education and Research Center (APDP-ERC), Lisbon, Portugal; g Department of Medical Sciences, University of Aveiro, Portugal; h Molecular Bioscience PhD Programme, Instituto de Tecnologia Química e Biológica António Xavier, Universidade Nova de Lisboa, ITQB-NOVA, Oeiras, Portugal

## Abstract

Insulin-degrading enzyme (IDE) degrades and inactivates bioactive peptides such as insulin. As insulin is a master regulator of glucose homeostasis, lack of IDE is expected to have a profound impact on both insulin and glucose levels. This article shares data on glucose and insulin homeostasis of control, heterozygous and knockout mice for *Ide* after 18 weeks of a normal chow diet. This data article is related to a research article entitled “Knockout of insulin-degrading enzyme leads to mice testicular morphological changes and impaired sperm quality” (Meneses et al., 2019).

**Table:** Specifications Table

Subject area	*Medicine*
More specific subject area	*Endocrinology, Diabetes and Metabolism*
Type of data	*Graphs of analyzed data*
How data was acquired	*Insulin Concentration was assessed using an Insulin ELISA kit* *Blood glucose levels during Glucose Tolerance Test and Insulin Tolerance Test were measured with a Glucose meter*
Data format	*Analyzed*
Experimental factors	*Wild type, heterozygous and knockout mice for Ide fed with normal chow diet for 18 weeks.*
Experimental features	*All parameters were measured after 18 weeks of normal chow diet.* *Glucose tolerance test was performed at a dose of 1.*5 g/Kg *Insulin Tolerance test was performed at a dose of 0.5 UI/Kg*
Data source location	*Lisbon, Portugal*
Data accessibility	*Data is included in this data article*
Related research article	Meneses MJ, Borges DO, Dias TR, Martins FO, Oliveira PF, Macedo MP, Alves MG 2019. Knockout of insulin-degrading enzyme leads to mice testicular morphological changes and impaired sperm quality, Molecular and Cellular Endocrinology. 486:11–17.

**Table:** 

**Value of the data** • The data shows the metabolic profile of *Ide* knockout mice at 18 weeks of age, always under normal chow diet. • The data present in this data article show that *Ide* knockout mice present increased glucose and insulin levels at 18 weeks of age, as well as increased insulin resistance. • Valuable for researchers interested in the impact of *Ide* deletion and insulin dysregulation, specifically hyperinsulinemia, on prediabetes onset. • This data article provides new insights about the role of IDE on glucose homeostasis and may be a basis for further studies aiming at unveiling the underlying mechanisms of prediabetes, namely due to primary hyperinsulinemia.

## 1 Data

**Fig. 1:**
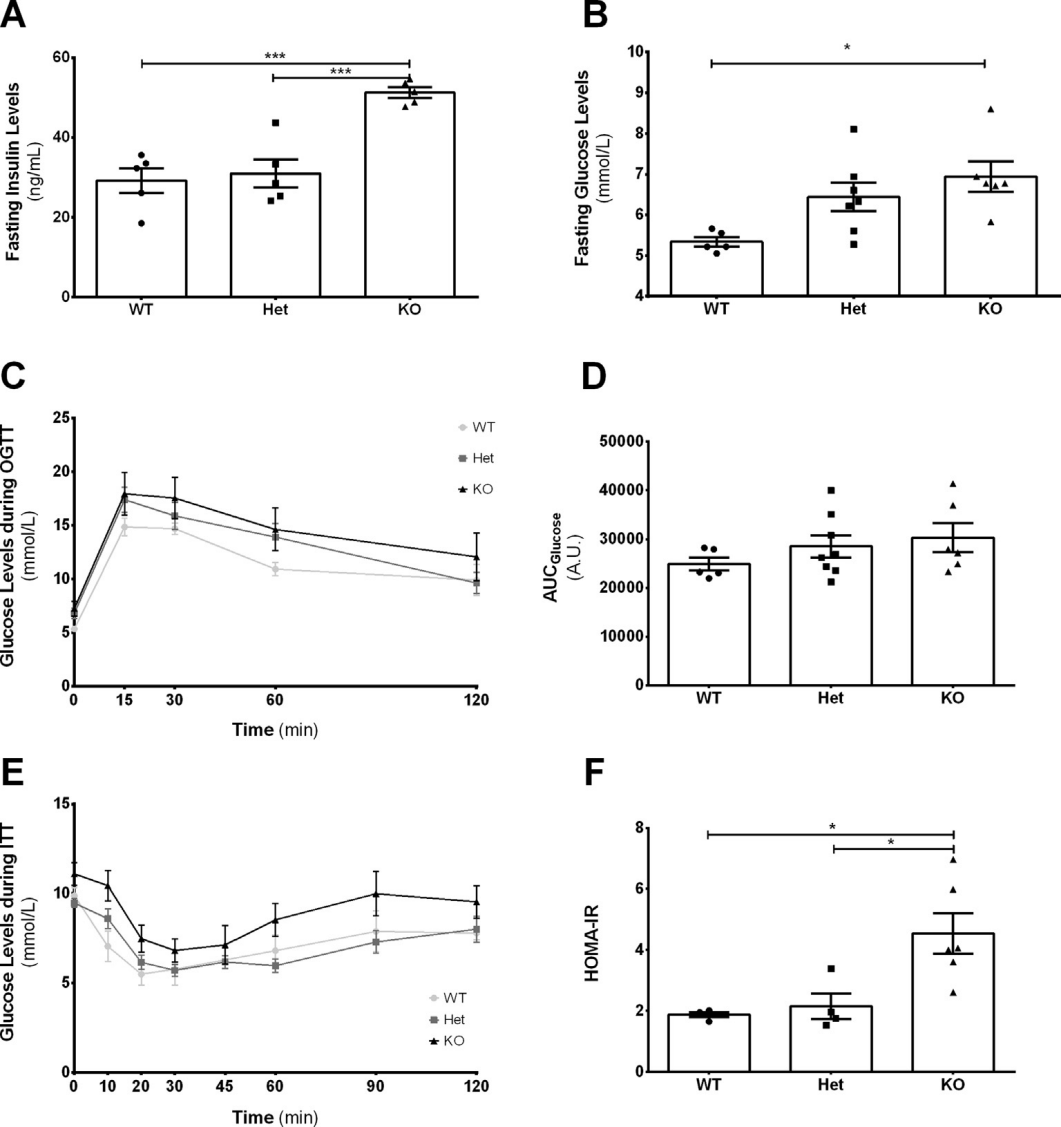
Effect of insulin-degrading enzyme on glucose and insulin homeostasis. The figure shows data of fasting insulin levels (panel A; see Table 1 for raw data), fasting glucose levels (panel B; see Table 2 for raw data), blood glucose levels during an oral glucose tolerance test (OGTT; panel C; see Table 3 for raw data), the area under the glucose curve during the OGTT (panel D; see Table 4 for raw data), blood glucose levels during an insulin tolerance test (ITT; panel E; see Table 5 for raw data) and the Homeostatic Model Assessment of Insulin Resistance (HOMA-IR; panel F; see Table 6 for raw data) of heterozygous (Het) or knockout (KO) mice for *Ide* and wildtype (WT) mice. Results are expressed as mean ± SEM (n = 5–8 for each condition). * − P < 0.05; ** − P < 0.01; *** − P < 0.001.

The data presented here are linked to a research article published separately by the same authors [1]. Here, we show the results regarding glucose and insulin fasting levels (Table 1, Table 2; Fig. 1A and B), glucose levels after a glucose bolus (Table 3; Fig. 1C), and the respective area under the curve (Table 4; Fig. 1D), which gives information about the capacity of the pancreas to release insulin in response to increased glucose levels [2]. Moreover, we also analyzed data regarding glucose levels after an insulin bolus (Table 5; Fig. 1E), and we have calculated the insulin resistance of these mice through the Homeostatic Model Assessment of Insulin Resistance (HOMA-IR; Table 6; Fig. 1F) [3].

**Table 1:** Fasting Blood Insulin Levels (ng/mL) of 18 weeks old wildtype (WT), heterozygous (Het) and knockout (KO) male mice for *Ide*. Corresponds to Fig. 1A. *** - p < 0.001 vs WT; ### - p < 0.001 vs Het.

Group	N1	N2	N3	N4	N5	Mean	SEM	p
WT	33.5	26.1	32.3	35.6	18.5	**29.20**	**3.11**	
Het	28.5	24.1	25.3	43.7	33.3	**30.98**	**3.56**	
KO	53.6	51.5	54.7	47.7	48.9	**51.28**	**1.33**	***
								**###**

The bold indicates the most important data of the table, as it shows the mean and the SEM.

**Table 2:** Fasting Blood Glucose Levels (mmol/L) of 18 weeks old wildtype (WT), heterozygous (Het) and knockout (KO) male mice for *Ide*. Corresponds to Fig. 1B. ns – non-significant; ** - p < 0.01 vs WT.

Group	N1	N2	N3	N4	N5	N6	Mean	SEM	p
WT	5.22	5.66	5.22	5.05	5.55	–	**5.34**	**0.256**	**ns**
Het	6.61	6.33	5.27	6.22	6.94	5.61	**6.16**	**0.621**	**ns**
KO	6.77	6.94	5.83	6.72	6.77	8.60	**6.94**	**0.907**	**

The bold indicates the most important data of the table, as it shows the mean and the SEM.

**Table 3:** Blood Glucose levels (mmol/L) during an oral glucose tolerance test (OGTT; 1.5 g glucose/Kg) of 18 weeks old wildtype (WT), heterozygous (Het) and knockout (KO) male mice for *Ide*. Corresponds to Fig. 1C. ns – non-significant.

Group	Time (min)				
	0	15	30	60	120
WT	5.22	11.99	13.32	10.38	7.77
	5.66	15.15	14.38	11.10	15.27
	5.22	15.21	13.99	10.55	7.44
	5.05	17.10	16.10	13.16	10.60
	5.55	14.77	15.65	9.38	8.44
Mean	**5.34**	**14.84**	**14.69**	**10.91**	**9.90**
SEM	**0.11**	**0.82**	**0.52**	**0.63**	**1.45**
Het	6.61	15.65	15.76	14.99	13.21
	6.33	16.93	15.15	11.99	9.16
	9.27	21.37	18.93	16.93	12.38
	8.10	22.98	23.15	20.43	12.43
	6.22	17.43	13.60	10.88	6.83
	6.94	17.32	15.27	12.16	9.60
	5.27	14.04	13.10	9.49	6.83
	5.61	13.43	12.05	14.32	6.49
Mean	**6.79**	**17.40**	**15.88**	**13.90**	**9.62**
SEM	**0.47**	**1.17**	**1.27**	**1.26**	**0.98**
KO	6.77	21.48	22.37	18.76	19.26
	6.94	15.93	19.98	17.04	18.15
	11.38	28.59	26.53	25.20	21.15
	5.83	14.10	17.15	15.04	7.72
	6.72	10.49	8.72	7.66	6.05
	6.77	20.32	17.04	11.21	8.88
	4.77	13.82	14.93	10.88	7.55
	8.60	18.76	13.60	11.10	7.72
Mean	**7.22**	**17.94**	**17.54**	**14.61**	**12.06**
SEM	**0.71**	**2.00**	**1.94**	**1.99**	**2.22**
p	**ns**	**ns**	**ns**	**ns**	**ns**

The bold indicates the most important data of the table, as it shows the mean and the SEM.

**Table 4:** Area under the curve (AUC) during an oral glucose tolerance test (OGTT; 1.5 g glucose/Kg) of 18 weeks old wildtype (WT), heterozygous (Het) and knockout (KO) male mice for *Ide*. Corresponds to Fig. 1D. ns – non-significant.

Group	N1	N	N3	N4	N5	N6	N7	N8	Mean	SEM	p
WT	21960	27938	23055	28223	23250	–	–	–	**24885**	**1324**	**ns**
Het	30803	26243	35115	39968	23573	26850	21203	24390	**28518**	**2247**	**ns**
KO	41408	36968	27915	27203	23333	24915	–	–	**30290**	**2948**	**ns**

The bold indicates the most important data of the table, as it shows the mean and the SEM.

**Table 5:** Blood glucose levels (mmol/L) during an Insulin Tolerance test (ITT; 0.5 UI/Kg) of 18 weeks old wildtype (WT), heterozygous (Het) and knockout (KO) male mice for *Ide*. Corresponds to Fig. 1E. ns – non-significant.

Group	Time (min)						
	0	10	20	30	60	90	120
WT	10.77	8.72	6.38	5.83	7.27	9.10	7.72
	9.27	6.55	5.77	7.27	8.05	8.94	8.49
	9.60	5.88	4.33	4.22	5.11	5.61	7.16
Mean	**9.88**	**7.05**	**5.50**	**5.77**	**6.81**	**7.88**	**7.79**
SEM	**0.45**	**0.85**	**0.61**	**0.88**	**0.88**	**1.14**	**0.39**
Het	9.33	8.49	5.16	5.00	5.50	7.60	8.33
	8.10	6.33	4.50	4.27	4.22	4.77	6.22
	9.66	10.77	6.61	6.49	6.22	8.44	8.38
	9.83	10.38	8.05	6.66	7.27	10.66	13.16
	8.94	8.10	5.66	5.61	6.22	6.05	6.11
	10.44	6.77	5.50	6.05	5.77	6.66	7.55
	9.05	8.55	7.16	6.77	7.83	9.38	8.55
	9.33	7.33	5.27	4.77	5.38	5.66	7.38
Mean	**9.33**	**8.34**	**5.99**	**5.70**	**6.05**	**7.40**	**8.21**
SEM	**0.24**	**0.56**	**0.42**	**0.33**	**0.40**	**0.71**	**0.78**
KO	12.77	13.54	7.49	7.77	10.38	12.21	12.77
	11.88	8.72	5.22	5.16	7.55	9.10	8.66
	10.88	9.55	6.83	6.00	7.05	8.27	11.38
	11.93	12.38	8.60	9.21	11.71	10.66	8.94
	8.33	10.10	10.49	7.33	8.94	14.16	9.05
	10.82	8.38	6.22	5.44	5.55	5.55	6.44
Mean	**11.10**	**10.45**	**7.48**	**6.82**	**8.53**	**9.99**	**9.54**
SEM	**0.63**	**0.85**	**0.76**	**0.64**	**0.93**	**1.24**	**0.91**
p	**ns**	**ns**	**ns**	**ns**	**ns**	**ns**	**ns**

The bold indicates the most important data of the table, as it shows the mean and the SEM.

**Table 6:** HOMA-IR (Homeostatic Model Assessment for Insulin Resistance) of 18 weeks old wildtype (WT), heterozygous (Het) and knockout (KO) male mice for *Ide*. Corresponds to Fig. 1F. * - p < 0.05 vs WT; # - p < 0.05 vs Het.

Group	N1	N2	N3	N4	N5	N6	Mean	SEM	p
WT	1.96	1.65	1.88	2.01	–	–	**1.88**	**0.079**	
Het	1.53	1.76	3.39	1.96	–	–	**2.16**	**0.419**	
KO	4.06	4.00	6.96	5.99	3.61	2.61	**4.54**	**0.660**	*
									**#**

The bold indicates the most important data of the table, as it shows the mean and the SEM.

## 2 Experimental design, materials, and methods

### 2.1 Animals

Full body *Ide* heterozygous C57BL6/N mice were acquired from the European Mouse Mutant Archive (EMMA). After heterozygous breeding, wild type (WT), heterozygous (Het) and knockout (KO) mice were generated and maintained on a 12 h light/dark cycle with standard chow diet (Special Diets Services, United Kingdom) and water *ad libitum* at Instituto Gulbenkian de Ciência (Oeiras, Portugal). Mice were monitored for body weight and blood glucose levels and all procedures followed ARRIVE guidelines and the Europeans laws (Directive 2010/63/EU) regarding the use of animals in research.

### 2.2 Oral glucose tolerance test

At 18 weeks old, and after an overnight fast, blood was collected from the mouse tail to measure blood glucose and insulin levels using a glucose meter and a mouse insulin ELISA kit (CrystalChem, Illinois, USA), respectively. Blood glucose levels were also measured 15, 30, 60 and 120 min after oral glucose administration (1.5 g/kg).

### 2.3 Insulin tolerance test

At 18 weeks old, and after 5 h of fasting, blood glucose levels were measured using a glucose meter before and 10, 20, 30, 45, 60, 90 and 120 min after insulin intraperitoneal injection (0.5 UI/kg).

### 2.4 Statistical analysis

The statistical significance among the experimental groups was assessed by one-way ANOVA. Experimental data is shown as mean ± SEM. Statistical analysis was performed using GraphPad Prism 6 (GraphPad software, San Diego, CA, USA). p < 0.05 was considered significant.

## References

[1] Meneses M.J., Borges D.O., Dias T.R., Martins F.O., Oliveira P.F., Macedo M.P., Alves M.G. (2019). Knockout of insulin degrading enzyme leads to mice testicular morphological changes and impaired sperm quality. Mol. Cell. Endocrinol..

[2] Bartoli E., Fra G.P., Schianca G.P.C. (2011). The oral glucose tolerance test (OGTT) revisited. Eur. J. Intern. Med..

[3] Matthews D.R., Hosker J.P., Rudenski A.S., Naylor B.A., Treacher D.F., Turner R.C. (1985). Homeostasis model assessment: insulin resistance and beta-cell function from fasting plasma glucose and insulin concentrations in man. Diabetologia.

