# Supplementary material for: Data on metabolic profile of insulin-degrading enzyme knockout mice
Source: Data Brief. 2019 May 24;25:104023. doi: 10.1016/j.dib.2019.104023 (PMC6557727; doi:10.1016/j.dib.2019.104023)
Supplement: Supplementary file 1 — Multimedia component 1 [file mmc1.docx]

**Conflict of Interest Form**

All the co-authors declare that there is no conflict of interest regarding the publication of this manuscript.
